# Supplementary material for: Causal Relationship between Aging and Anorexia Nervosa: A White-Matter-Microstructure-Mediated Mendelian Randomization Analysis
Source: Biomedicines. 2024 Aug 16;12(8):1874. doi: 10.3390/biomedicines12081874 (PMC11351342; doi:10.3390/biomedicines12081874)
Supplement: Supplementary file 1 [file biomedicines-12-01874-s001.zip › Supplementary Figure/Figure S4/Singe SNP plot of MR analysis for TL to ubm-b-1613.pdf]

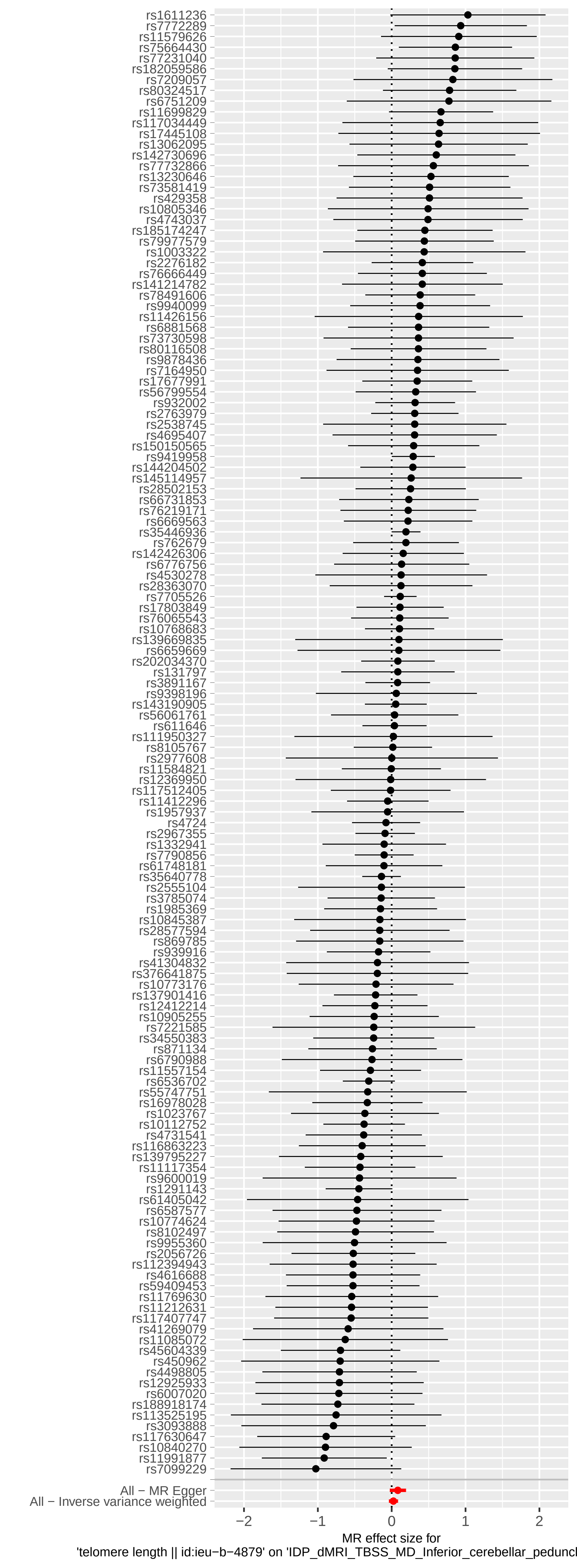

rs1611236
rs7772289
rs11579626
rs75664430
rs77231040
rs182059586
rs7209057
rs80324517
rs6751209
rs11699829
rs117034449
rs17445108
rs13062095
rs142730696
rs77732866
rs13230646
rs73581419
rs429358
rs10805346
rs4743037
rs185174247
rs79977579
rs1003322
rs2276182
rs76666449
rs141214782
rs78491606
rs9940099
rs11426156
rs6881568
rs73730598
rs80116508
rs9878436
rs7164950
rs17677991
rs56799554
rs932002
rs2763979
rs2538745
rs4695407
rs150150565
rs9419958
rs144204502
rs145114957
rs28502153
rs66731853
rs76219171
rs6669563
rs35446936
rs762679
rs142426306
rs6776756
rs4530278
rs28363070
rs7705526
rs17803849
rs76065543
rs10768683
rs139669835
rs6659669
rs202034370
rs131797
rs3891167
rs9398196
rs143190905
rs56061761
rs611646
rs111950327
rs8105767
rs2977608
rs11584821
rs12369950
rs117512405
rs11412296
rs1957937
rs4724
rs2967355
rs1332941
rs7790856
rs61748181
rs35640778
rs2555104
rs3785074
rs1985369
rs10845387
rs28577594
rs869785
rs939916
rs41304832
rs376641875
rs10773176
rs137901416
rs12412214
rs10905255
rs7221585
rs34550383
rs871134
rs6790988
rs11557154
rs6536702
rs55747751
rs16978028
rs1023767
rs10112752
rs4731541
rs116863223
rs139795227
rs11117354
rs9600019
rs1291143
rs61405042
rs6587577
rs10774624
rs8102497
rs9955360
rs2056726
rs112394943
rs4616688
rs59409453
rs11769630
rs11212631
rs117407747
rs41269079
rs11085072
rs45604339
rs450962
rs4498805
rs12925933
rs6007020
rs188918174
rs113525195
rs3093888
rs117630647
rs10840270
rs11991877
rs7099229
All − MR Egger
All − Inverse variance weighted
−2
−1
0
1
2
MR effect size for
'telomere length || id:ieu−b−4879' on 'IDP_dMRI_TBSS_MD_Inferior_cerebellar_peduncl
